# Detection of bla NDM-1 Encoding Imepenemase among the Imipenem-Resistant Gram-Negative Bacilli Isolated from Various Clinical Samples at a Tertiary Care Hospital of Eastern Nepal: A Descriptive Cross-Sectional Study

**DOI:** 10.1155/2020/8861204

**Published:** 2020-07-27

**Authors:** S. Gautam, N. R. Bhattarai, K. Rai, A. Poudyal, B. Khanal

**Affiliations:** Department of Microbiology and Infectious Diseases, B. P Koirala Institute of Health Sciences (BPKIHS), Dharan, Sunsari 56700, Nepal

## Abstract

**Background:**

Carbapenem resistance among Gram-negative isolates caused by the production of the metallo-*β*-lactamase (MBL) enzyme is being increasingly reported worldwide. One of the newly emerged metallo-*β*-lactamases is New Delhi metallo-*β*-lactamase. Data regarding its occurrence in hospital setting and percentage prevalence among different Gram-negative bacterial isolates are lacking in our part. This study has been undertaken for determining the presence of the bla NDM-1 gene among the clinical isolates of imipenem-resistant Gram-negative bacteria in a tertiary care center in Dharan, Nepal.

**Methods:**

A total of 75 imipenem-resistant Gram-negative isolates were studied. These were screened for metallo-*β*-lactamase (MBL) production by phenotypic assays such as double-disc synergy test (DDST) and combined disc diffusion test (CDDT). PCR was performed for the molecular detection of gene NDM-1. Ten-disc method was performed to detect the presence of ESBL, AmpC, carbapenamase, and K1 *β*-lactamase production.

**Results:**

Using the molecular method, bla NDM-1 was detected in 36% of the isolates. Phenotypically, double-disc synergy test (DDST) and combined disc diffusion test (CDST) detected MBL production in 38.7% and 37.3% of the isolates, respectively. Ten-disc method detected ESBL in 26.6% of the isolates, but none of the isolates was found to be AmpC, carbapenamase, and K1 *β*-lactamase producers.

**Conclusion:**

A high percentage of the NDM-1 producer was noted among imipenem-resistant GNB. Apart from performing only antimicrobial sensitivity test, phenotypic and molecular screening should be employed to find out the actual number of metallo-*β*-lactamase producers and the existence of the resistance gene.

## 1. Background

Carbapenems are considered as the last resort of treatment for infection caused by MDR Gram-negative bacilli [1]. Resistance even to these groups of drugs has made treatment difficult. One of the mechanisms of resistance to beta-lactam is by the production of the *β*-lactamase enzyme. New enzymes evolve over time by mutation in the existing resistance enzyme in response to environmental pressure. Nowadays, metallo-*β*-lactamase enzyme is a major public health concern because of its rapid dissemination worldwide.

Metallo-*β*-lactamases are enzymes that break down beta-lactam drugs. NDM is a type of MBL produced by the bacteria that makes it resistant to a broad range of beta-lactam antibiotics including the carbapenem group except aztreonam [2].

NDM-1 has been identified both in the bacterial chromosome and plasmid. Plasmids carrying the bla NDM-1 gene also carry a number of other genes conferring resistance to many more classes of drugs apart from beta-lactam as aminoglycoside, macrolide, and sulfamethoxazole. Thus, NDM-producing strains are resistant to almost all classes of drugs available [3].

Presence of the NDM producer in any hospital setting is an alarming situation as plasmids present in such strains are easily transferrable, also capable of wide rearrangement that points toward its widespread horizontal transfer. These strains may be present in people as a carrier not producing any symptoms. There is a lack of routine standardized phenotypic test for its detection [4].

This study was conducted to find out the resistant mechanism in carbapenem-resistant Gram-negative bacteria phenotypically and to detect resistance gene NDM-1 in our hospital setting.

## 2. Methods

A descriptive cross-sectional study was conducted at the Department of Microbiology, B. P. Koirala Institute of Health Sciences, from September 2015 to July 2016. Seventy-five imipenem-resistant isolates from various clinical samples such as blood, body fluid, endotracheal tube, central venous catheter tips (CVP tips), pus, tissue, and urine submitted to the microbiology laboratory for routine culture and sensitivity testing were included in the study.

Gram-negative bacteria were identified with reference to colony morphology, Gram staining, catalase test, and oxidase test. Further speciation was done on the basis of different biochemical tests performed as per the standard microbiological guidelines [5]. Antibiotic sensitivity testing was performed on Mueller–Hinton agar (MHA) according to CLSI guidelines [6].

### 2.1. Combined Disc Diffusion Test

After swabbing the test organism over the entire MHA plate, two imipenem (10 *μ*g) disks, one containing 10 *μ*l of 0.5 M (750 *μ*g) anhydrous EDTA, were placed 25 mm apart from center to center. An increase in the zone diameter of >7 mm around the IMP-EDTA disk compared to that of the IPM disk alone was considered positive for the MBL production [7].

### 2.2. Double-Disc Synergy Test

The test organism was inoculated on MHA as mentioned above. Two discs were applied 20 mm apart from center to center, one imipenem (10 *μ*g) disc and another blank disc to which 10 *μ*l of 0.5 M EDTA was added. Enhancement of the zone of inhibition in the area between imipenem and EDTA disc in comparison to the zone size on the far side of the drug was considered as a positive result [7].

### 2.3. Detection of the bla NDM-1 Gene

DNA was extracted from all the isolates by the boiling method [8], and 1 *μ*l of the isolated DNA was subjected to PCR with specific primers: NDM-1 forward (5`- CAGCACACTTCCTATCTC) and NDM-1 reverse (5`-GTAGTGCTCAGTGTCGGCAT). PCR was carried out in Eppendorf mastercycler ProS (Eppendorf, Germany) with the following conditions: initial denaturation for 5 min at 95°C followed by 35 cycles at 94° for 30 sec and then at 72°C for 30 sec. Final extension was for 10 min at 72°C, and the PCR product was kept in 4°C [9].

Agarose gel electrophoresis was performed for the PCR product. Staining was done with ethidium bromide solution. For visualization, agar was kept in a transilluminator gel box called the G box. PCR amplicon appeared as bands on the gel which was captured in the presence of UV light by the computer system [10]. The expected PCR amplicon size was 294 bp [9].

### 2.4. Detection of ESBL

Detection of ESBL was done as per the clinical laboratory standard institute- (CLSI-) recommended method. Combined disc method was followed using antimicrobial discs (HiMedia, Mumbai, India)—cefotaxime (30 *μ*g), ceftazidime (30 *μ*g), cefotaxime-clavulanic acid (30/10 *μ*g), and ceftazidime-clavulanic acid (30/10 *μ*g). The test organism was inoculated on the MHA plate using a lawn culture. Each disc was kept at least 20 mm apart, center to center, on the MHA plate and incubated overnight at 35°C [11].

Isolates resistant to cefotaxime and ceftazidime but sensitive to cefotaxime-clavulanic acid and ceftazidime clavulanic acid with an enhanced zone of inhibition ≥5 mm was confirmed as the ESBL producer.

### 2.5. Ten-Disc Diffusion Method for the Screening of Various Resistance Mechanisms

Ten discs were placed on the surface of the 150 mm MHA plate after inoculation of the test organism. After overnight incubation, each plate was examined for the zone of inhibition. The diameter of the zone of inhibition was interpreted as sensitive, intermediate, and resistant.

Antimicrobials used for the test were as follows:

  Ertapenem (10 *μ*g)
  Ceftazidime (30 *μ*g)
  Ceftazidime/clavulanic acid (30/10 *μ*g)
  Imipenem (10 *μ*g)
  Cefotaxime (30 *μ*g)
  Cefotaxime/clavulanic acid (30/10 *μ*g)
  Aztreonam (10 *μ*g)
  Cefoxitin (30 *μ*g)
  Ceftriaxone (30 *μ*g)
  Cefepime (30 *μ*g)

### 2.6. Detection of the AmpC Producer [12]

Isolates resistant to cefoxitin (zone of inhibition ≤18 mm) but sensitive to cefepime (zone of inhibition ≥ 18 mm) indicate AmpC producers.

### 2.7. Detection of K1 *β*-Lactamase [13]

A strain was considered as a K1 *β*-lactamase producer if it was resistant to aztreonam (zone of inhibition ≤ 27 mm) and ceftriaxone (zone of inhibition ≤ 25 mm) and sensitive to cefotaxime (zone of inhibition ≥26 mm) and ceftazidime (zone of inhibition ≥ 21 mm).

### 2.8. Detection of Carbapenamase [14]

For the screening of carbapenamase, a strain should be resistant to ertapenem (zone of inhibition ≤ 22 mm) and sensitive to imipenem (zone of inhibition ≥ 23 mm).

### 2.9. Statistical Analysis

The data were entered into Microsoft Excel 2013 and analyzed by using SPSS version 16 (SPSS Inc., Chicago, IL, USA). Kappa agreement tool was applied for the measurement of agreement between two phenotypic methods for MBL detection and PCR.

## 3. Results

Seventy-five imipenem-resistant Gram negative bacilli isolated from various clinical specimens were included in the study. The total number of different Gram-negative bacilli obtained and their antimicrobial resistance pattern are shown in Table 1.

**Figure 1:**
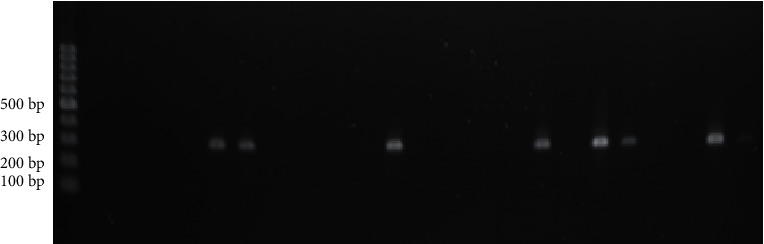
Agarose gel showing the PCR-amplified product of the bla NDM-1 gene.

Out of 75 obtained isolates, 38 (50.6%) were positive for MBL production by the combined result of both EDTA-based tests (CDDT and DDST) as depicted in Table 2. Highest MBL producers were *E. coli* 11 (68.8%) followed by *K. pneumoniae* 8 (50%) and *A. anitratus* 16 (45.7%). MBL-positive isolates were from ward 15 (39.47%), ICU 8 (21.05%), OPD 8 (21.05%), and emergency 7 (18.42%). Twenty-seven isolates (36%) were positive for resistance gene bla NDM-1 by PCR. PCR-amplified product of the bla NDM-1 gene is shown in Figure 1.

Ten-disc method was performed to study other resistance mechanisms apart from MBL. ESBL production was noted in 20 (26.6%) isolates using the ten-disc method. Rate of ESBL production differed in various GNB as depicted in Table 3. None of the isolates was found to be AmpC, K1 *β*-lactamase, and carbapenamase producers.

## 4. Discussion

In our study, MBL producers formed 50.6% of the total isolates as detected by using two phenotypic methods. Two different phenotypic methods, i.e., combined disc diffusion test and double-disc synergy test, were performed to detect MBL, whereas PCR was carried out to find the presence of the bla NDM-1 gene. Concordance between combined disc test and PCR as well as double-disc test and PCR was measured using the kappa agreement tool.

Kappa value between PCR and combined disc was 0.627 denoting good agreement, whereas it was 0.374, a fair agreement between PCR and double-disc test. This indirectly gives evidence that DDST and CDDT are not equally sensitive for the detection of MBL which is supported by the report of Panchal et al. [15]. Furthermore, it has been tested that results of MBL detection tests not only depend upon the methodology employed but also on the beta-lactam drugs employed, MBL inhibitor substance used, and bacterial genus tested [16].

Our study showed varying rate of MBL production in various GNB. Highest MBL production was seen in *E. coli* (68.8%) followed by *K. pneumoniae* (50%) and *A. anitratus* (45.7%). This finding was higher than that reported in the previous study from Nepal [17].

Imipenem-resistant isolates were resistant to almost all antibiotics tested. Thus, sensitivity to tigecycline and colistin was tested further using the disc-diffusion method. Highest rate of resistance to tigecycline was seen with *Pseudomonas aeruginosa* (100%) followed by *Acinetobacter anitratus* (51.4%), *E. coli* (31.25%), and *Klebsiella pneumoniae* (12.5%).

In our study, all Gram-negative bacilli were found to be sensitive to colistin by the disc-diffusion method except *Pseudomonas aeruginosa* which showed 100% resistance to colistin. Resistance to colistin has been documented in different parts of the world. A study conducted in a tertiary care hospital in Pakistan detected 3 colistin-resistant strains out of 885 isolates from hospitalized patients among which 2 were *E. coli* and 1 was *Acinetobacter* species [18]. This is a matter of concern as with the development of resistance even to these “reserve” drugs, we will be left with crisis of antibiotics in the future.

As plasmid-carrying bla NDM frequently carries AmpC or ESBL gene, search for other resistance mechanisms along with NDM was also attempted. In the present study, ESBL production was noted in 26.6% of the total imipenem-resistant isolates. Highest producer was found to be *K. pneumoniae* (62.5%) followed by *E*. *coli* (12.5%) in contrast to the results of the studies conducted at our center in the past by Shrestha et al. taking into account clinical isolates causing pyogenic infection which demonstrated highest ESBL production in *E. coli* followed by *K. pneumoniae* [19]. Similarly, Manipal Teaching Hospital, Pokhara, Nepal, also reported higher frequency of ESBL-producing *E*. *coli* (81.6%) than *K. pneumoniae* (4.1%). In contrary to this, Khanal et al. in their study found higher rate of ESBL production in 42.8% of *P. aeruginosa* followed by K. *pneumoniae* (34.3%) [20].

Detection of AmpC or ESBL was done by phenotypic methods. Out of the total isolates which tested positive for bla NDM-1 gene, 37% were found to be ESBL producers. This result was in agreement with that observed by Rahseed et al., where along with NDM, other *β*-lactamase genes such as AmpC and ESBL were found in a single bacterial strain [21].

We did not encounter any AmpC- and K1 *β*-lactamase-producing Gram-negative bacilli in our study.

## 5. Conclusion

The study suggested that MBL production in 50.6% and presence of bla NDM-1 in 36% of the clinically significant Gram-negative isolates are quite alarming. Circulation of bla NDM-1 in our hospital setting in such a high rate demands immediate implementation of strong infection control practices so that its further dissemination is checked.

With this study, an attempt has been made to study the distribution of resistant Gram-negative clinical isolates in our setting. Clinical microbiology laboratory has a role to play in early detection of resistant isolates—an initial but crucial step towards the control of occurrence and spread of these resistant pathogens.

## Figures and Tables

**Table 1:** Antimicrobial resistance pattern among various Gram-negative isolates (*n* (%)).

Antibiotics	*Acinetobacter anitratus* (*n* = 35)	*Escherichia coli* (*n* = 16)	*Klebsiella pneumoniae* (*n* = 16)	*Pseudomonas aeruginosa* (*n* = 5)	*Citrobacter koseri* (*n* = 2)	*Enterobacter aerogenes* (*n* = 1)
Ciprofloxacin	35 (100%)	16 (100%)	16 (100%)	5 (100%)	1 (50%)	1 (100%)
Amikacin	13 (81.25%)	29 (82.85%)	16 (100%)	5 (100%)	2 (100%)	0 (0%)
Gentamicin	14 (87.5%)	33 (94.2%)	16 (100%)	5 (100%)	1 (50%)	1 (100%)
Ampicillin	35 (100%)	16 (100%)	16 (100%)	5 (100%)	2 (100%)	1 (100%)
Cefotaxime	35 (100%)	16 (100%)	16 (100%)	5 (100%)	2 (100%)	1 (100%)
Cefepime	35 (100%)	16 (100%)	16 (100%)	5 (100%)	2 (100%)	1 (100%)
Imipenem	35 (100%)	16 (100%)	16 (100%)	5 (100%)	2 (100%)	1 (100%)
Tigecycline	18 (51.4%)	5 (31.25%)	2 (12.5%)	5 (100%)	1 (50%)	1 (100%)
Colistin	0 (0%)	0 (0%)	0 (0%)	5 (100%)	0 (0%)	0 (0%)

**Table 2:** Percentage of MBL detected phenotypically and bla NDM-1 detected by PCR.

Organism	MBL detected by phenotypic methods (CDDT and DDST)	bla NDM-1 detected by PCR
*A. anitratus* (*n* = 35)	16 (45.7%)	13 (37.14%)
*K. pneumoniae* (*n* = 16)	8 (50%)	7 (43.75%)
*E. coli* (*n* = 16)	11 (68.8%)	4 (25%)
*P. aeruginosa* (*n* = 5)	1 (20%)	2 (40%)
*E. aerogenes* (*n* = 1)	1 (100%)	1 (100%)
*C. koseri* (*n* = 2)	1 (50%)	0 (0%)

**Table 3:** ESBL production in various Gram-negative bacilli (*n* = 75).

S. no	Organism	ESBL producer
1	*A. anitratus* (*n* = 35)	7 (20%)
2	*K. pneumoniae* (*n* = 16)	10 (62.5%)
3	*E. coli* (*n* = 16)	2 (12.5%)
4	*P. aeruginosa* (*n* = 5)	0 (0%)
5	*E. aerogenes* (*n* = 1)	1 (100%)
6	*C. koseri* (*n* = 2)	0 (0%)
	Total = 75	20 (26.6%)

## Data Availability

The data used to support the findings of this study are available from the corresponding author upon request.

## Abbreviations

NDM: New Delhi metallo-*β*-lactamase
CLSI: Clinical and Laboratory Standards Institute
EDTA: Ethylenediaminetetraacetic acid
MBL: Metallo-*β*-lactamase
ESBL: Extended-spectrum *β*-lactamase.
